# Quantifying Two-Dimensional Surface Displacements Using High-Resolution Cosmo-SkyMed, TerraSAR-X and Medium-Resolution Sentinel-1 SAR Interferometry: Case Study for the Tengiz Oilfield

**DOI:** 10.3390/s22176416

**Published:** 2022-08-25

**Authors:** Emil Bayramov, Giulia Tessari, Martin Kada

**Affiliations:** 1 School of Mining and Geosciences, Nazarbayev University, 53 Kabanbay Batyr Ave, Block 6, Room 6510, Nur-Sultan City 010000, Kazakhstan; 2 Institute of Geodesy and Geoinformation Science, Technische Universität Berlin, Kaiserin-Augusta-Allee 104-102, KAI 2302, 10553 Berlin, Germany; 3 Sarmap SA, Via Stazione 52, 6987 Caslano, Switzerland

**Keywords:** remote sensing, oil reservoir, land deformations, SAR, InSAR, SBAS, Sentinel-1, Cosmo-SkyMED, TerraSAR-X

## Abstract

The present study was aimed at comparing vertical and horizontal surface displacements derived from the Cosmo-SkyMED, TerraSAR-X and Sentinel-1 satellite missions for the detection of oil extraction-induced subsidence in the Tengiz oilfield during 2018–2021. The vertical and horizontal surface displacements were derived using the 2D decomposition of line-of-sight measurements from three satellite missions. Since the TerraSAR-X mission was only available from an ascending track, it was successfully decomposed by combining it with the Cosmo-SkyMED descending track. Vertical displacement velocities derived from 2D Decomposition showed a good agreement in similar ground motion patterns and an average regression coefficient of 0.98. The maximum average vertical subsidence obtained from the three satellite missions was observed to be −57 mm/year. Higher variations and deviations were observed for horizontal displacement velocities in terms of similar ground motion patterns and an average regression coefficient of 0.80. Fifteen wells and three facilities were observed to be located within the subsidence range between −55.6 mm/year and −42 mm/year. The spatial analyses in the present studies allowed us to suspect that the subsidence processes occurring in the Tengiz oilfield are controlled not solely by oil production activities since it was clearly observed from the detected horizontal movements. The natural tectonic factors related to two seismic faults crossing the oilfield, and terrain characteristics forming water flow towards the detected subsidence hotspot, should also be considered as ground deformation accelerating factors. The novelty of the present research for Kazakhstan’s Tengiz oilfield is based on the cross-validation of vertical and horizontal surface displacement measurements derived from three radar satellite missions, 2D Decomposition of Cosmo-SkyMED descending and TerraSAR-X ascending line-of-sight measurements and spatial analysis of man-made and natural factors triggering subsidence processes.

## 1. Introduction

Spaceborne SAR interferometry is a remote sensing technique used for measuring ground displacement and has been intensively used by petroleum, gas and mining industries for many years [1,2,3,4,5,6,7,8,9,10,11]. The study area of the present research is the Tengiz oilfield located at the coast of the Caspian Sea. It is well-known that oil and gas exploitation and injection activities lead to ground deformations in oil and gas fields. This may cause geological hazards and consequent damages to oil and gas infrastructure [12,13,14]. Additionally, reservoir volumetric changes may highlight reservoir compartmentalization and fault reactivation [15,16,17].

Interferometric Synthetic Aperture SAR (InSAR) plays an important role in determining surface displacements induced by fluid extraction and injection for modeling of reservoir dynamic behavior and performs necessary improvements to achieve more effective exploitation and geohazards’ risk assessment principles [16,17]. The studies by Ferretti et al. [4], Del Conte et al. [16], Rocca et al. [17] and Even et al. [18] clearly state that dense and highly precise vertical and horizontal displacement are critical information for regular reservoir monitoring, characterization and geomechanical analysis.

The ongoing subsidence affecting the Tengiz oilfield is confirmed by the studies of Comola et al. [19], Grebby et al. [20], Orynbassarova [21] and Bayramov et al. [22,23]. The studies by Grebby et al. [20] and Orynbassarova [21] used Envisat 2004–2009 and Sentinel-1 2016–2017 SAR satellite images to assess ground deformation of the Tengiz oilfield caused by oil extraction. Bayramov et al. [22] analyzed Sentinel-1 2018–2020 SAR satellite images acquired from ascending (ASC) and descending (DSC) tracks to perform 3D and 2D decompositions for vertical and horizontal surface displacements in the Tengiz oilfield. Del Conte et al. [16] and Comola et al. [19] applied Envisat and RADARSAT-1 images acquired between 2004 and 2007 to couple SAR measurements and 3D numerical modeling for Tengiz oil reservoir monitoring and geomechanical model calibration. Zhantaev et al. [24] and Zhantayev et al. [25] observed vertical displacements in the Tengiz oilfield using ENVISAT 2004–2009 and ALOS PALSAR 2007–2010 SAR satellite images. It is necessary to emphasize that all of these studies concluded with different annual vertical displacement velocities because of differences in used satellite missions, period of observations and applied interferometric techniques.

The present study is aimed at comparing vertical and horizontal surface displacements derived from the Cosmo-SkyMed (CSK), TerraSAR-X (TSX) and Sentinel-1 (SNT1) satellite missions for the detection of oil extraction-induced subsidence in the Tengiz oilfield during the period 2018–2021.

The final goal of this research is to contribute to the remote surveillance used by petroleum and gas industries worldwide by providing insights on the applicability of various high-resolution X-band SAR satellite missions in comparison to medium-resolution C-band acquisitions. Advantages of space-borne SAR applications for petroleum and gas industries compared to in-situ geodetic measurements are primarily the following: wide spatial coverage, availability of time-series observations, site accessibility, cost efficiency and safe remote measurements. However, it is necessary to consider the capability of InSAR application in terms of spatial and temporal resolution as well as the consequent possible measurements to decide the practical applicability for specific monitoring requirements. For instance, sometimes the role of in-situ geodetic measurements may be irreplaceable within oil and gas terminals or underground mining to perform necessary geodetic measurements with higher levels of precision. Hence, geodetic measurements on the ground are highly precise and suitable for detailed scales of measurements, but they are limited to the spatial coverage of observations and are not always safe [14]. Historical ground geodetic measurements of the Tengiz oilfield were not accessible in the framework of this study; therefore, it was decided to proceed with the cross-validation approach between independent datasets acquired by different satellite missions.

The research goals of the present studies are as follows:

1. Geospatial comparative data analytics and cross-validation of vertical and horizontal interferometric measurements derived from high-resolution CKS, TSX and medium-resolution SNT1 satellite missions;
2. Coupling of CSK and TSX SAR processing results for the decomposition of line-of-sight measurements to vertical and horizontal measurements;
3. Assessment of vertical and horizontal displacement velocities, cumulative vertical displacements and associated movement hotspots using CSK, TSX and SNT1 imagery acquired during the period 2018–2021.

The primary novelty of the present research for Kazakhstan is based on the cross-validation of vertical and horizontal surface displacement measurements derived from three radar satellite missions (CSK, TSX and SNT1) in the Tengiz oilfield. The secondary novelty is the combination of CKS descending and TSX ascending tracks to derive vertical and horizontal surface displacements. The third novelty for the Tengiz oilfield is the general spatial root cause analysis of subsidence processes in terms of man-made and natural surface displacement triggering factors.

As a result of these studies, it was possible to quantitatively assess relationships between vertical (uplift and subsidence) and horizontal (east–west) motions derived from CSK, TSX and SNT1 radar images for the Tengiz oilfield. This also allowed us to comparatively evaluate the role and reliability of these radar satellite missions in the identification of the spatial deformation patterns, subsidence and uplift hotspots for this oilfield.

The paper is organized as follows: introduction section describing previous surface displacement studies focused on the Tengiz oil field; research goals and novelties for the Tengiz oilfield; data processing section describing research area; applied satellite missions and interferometric data processing and geospatial analyses techniques; results section describing achieved research goals; discussion session describing achieved results and limitations of the present research; conclusion section describing a summary of the present research.

## 2. Study Area

**Figure 1:**
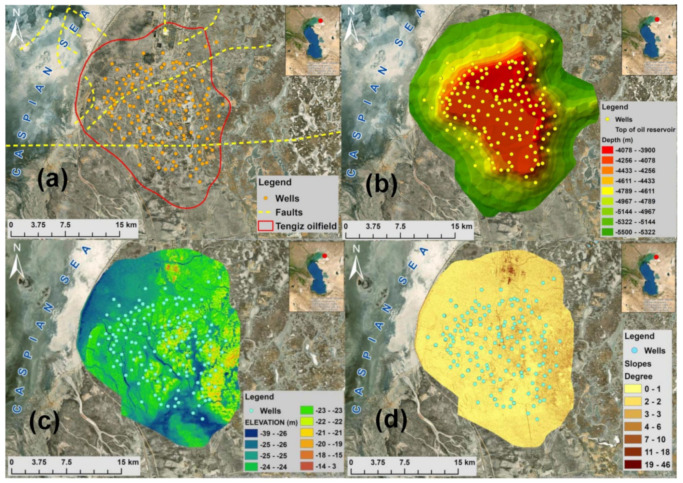
Tengiz oilfield: (**a**) representation of wells and faults; (**b**) top of the oil reservoir; (**c**) elevation; (**d**) slopes.

The Tengiz oilfield was discovered in 1979 and its development started in 1991. It is located in Atyrau oblast of Kazakhstan on the north-eastern coast of the Caspian Sea and covers an area of 2500 km^2^ extending 19 km in length and 21 km in width with up to 1.5 km thickness (Figure 1a). It is one of the largest and deepest oil fields in Kazakhstan with over 100 drilled wells [25]. The reservoir is a carbonate build-up from Devonian through Carboniferous age with an extensive natural fracture network [26]. According to Anissimov et al. [27], the Tengiz oilfield is intersected by faults associated with the active seismicity of this area (Figure 1a). The estimated resources of the reservoir are 25.5 billion oil barrels stored at the depth range of 3885–5117 m [20] with a current production value of 720,000 barrels per day. As it is possible to observe in Figure 1b, the top of the oil reservoir lies at a depth of about 4000 m. Figure 1c,d shows the terrain flatness of the Tengiz oilfield with naturally formed depressions by snow and rainfalls [20]. The average annual precipitation of the Tengiz oilfield is around 158 mm [22]. The mean annual air temperature is 11 °C. Tengiz field’s climate is semi-arid with a temperature range between −30 °C in winter and 40 °C in summer.

## 3. Data Processing

**Figure 2:**
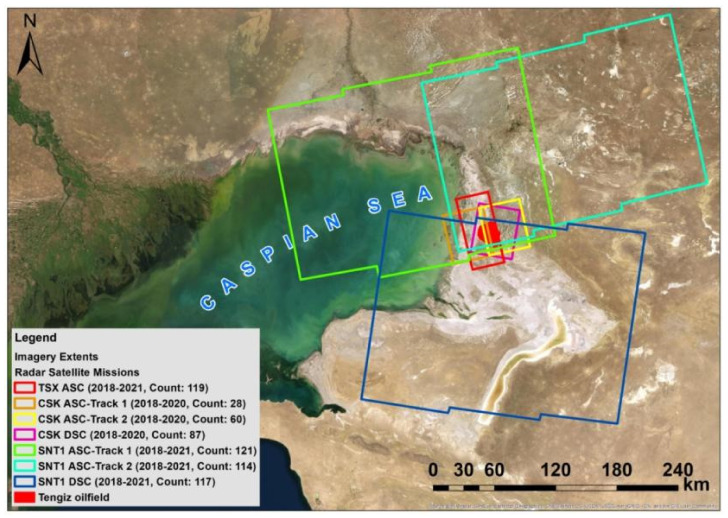
Extents, count and acquisition period of TSX, CSK and SNT1 imagery.

In this study, we used 119 TSX ASC SAR images from the German Aerospace Agency (DLR), 87 DSC and 88 CSK ASC SAR images from the Italian Space Agency (ASI), and 96 DSC and 235 ASC SNT1 SAR images from the European Space Agency (ESA). CSK SAR images covered a three-year span from January 2018 to December 2020, whereas TSX and SNT1 SAR images covered a four-year time span from January 2018 to December 2021. The high-resolution CSK and TSX SAR images have a spatial resolution of 3 m, making them optimal sources for the detection of detailed surface deformations within petroleum and gas fields. The SNT1 satellite mission has a medium resolution of 5 m by 20 m with free accessibility and larger spatial coverage per scene. TSX and CSK SAR images were available in HH polarization, while SNT1 in the VV ones. Both HH and VV polarizations are adequate for interferometric applications considering higher coherence and scattering aspects [28,29,30]. The characteristics of the used SAR images are presented in Table 1. The footprints of the CSK, TSX and SNT1 imagery are shown in Figure 2. CKS and SNT1 images fully covered the Tengiz oilfield whereas TSX had a minor gap from the eastern side of the oilfield.

**Figure 3:**
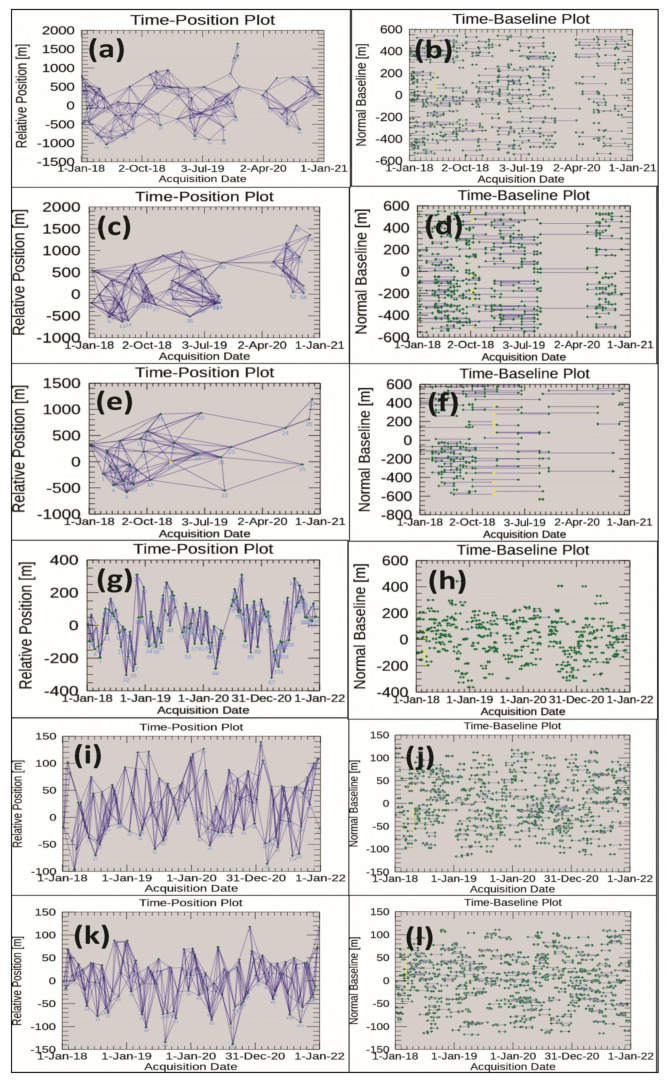
Connection graphs: time-position plot for SBAS and time-baseline plot for SBAS: (**a**,**b**) CSK DSC; (**c**,**d**) CSK ASC Right Track; (**e**,**f**) CSK ASC Left Track; (**g**,**h**) TSX ASC; (**i**,**j**) SNT1 DSC; (**k**,**l**) SNT1 ASC.

The connection graphs of the CSK, TSX and SNT1 images in Figure 3a–l show that all SAR images were well connected in time in order to perform interferometric processing over the period 2018–2021.

**Figure 4:**
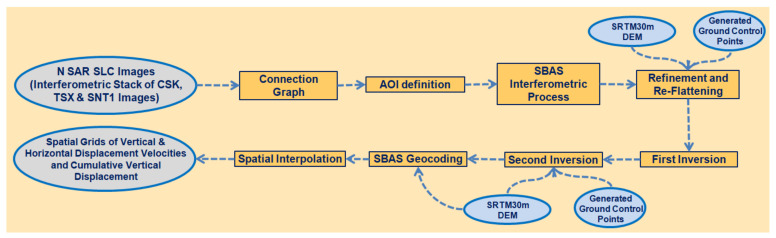
SBAS-InSAR interferometric processing workflow.

The TSX, CSK and SNT1 SAR images were processed using Small Baseline Subsets-Interferometric Synthetic Aperture SAR (SBAS-InSAR) techniques in the ENVI SARscape software version 5.6.2 (Sarmap SA, Via Stazione 52, 6987 Caslano, Switzerland) with the workflow presented in Figure 4 [33,34,35]. The multi-temporal SBAS technique has been selected as the most appropriate processing approach because of its ability to measure small deformations affecting distributed scatterers, low urbanized and low vegetated areas, characterized by spatially correlated deformation behaviors. The selection of the optimal pairs to be used is based on the minimization of the spatial and temporal baselines limiting temporal decorrelation effects [33,36,37,38,39,40]. In fact, the SBAS technique contributes to the provision of deformation results over areas characterized by low temporal coherence as in the case of the Tengiz oilfield.

For the **connection graph** stage, to reduce the geometrical and temporal decorrelation in further interferometric processing, a maximum temporal baseline of 180 days and a degree of redundancy of ten connections per scene were used. These allowed for the generation of 599 interferograms for the ASC track of TSX; 617 for DSC; 533 for left and 582 for right ASC tracks of CSK; and 547 for DSC; 511 for left and 499 for right ASC tracks of SNT1 datasets in the **SBAS-InSAR interferometric process** stage. A maximum perpendicular baseline of 500 m was used as criteria for TSX and CSK, while 200 m was set as the maximum normal baseline for SNT1 SAR images. The Shuttle Radar Topography Mission (SRTM) with a 30 m resolution was used as input for the topographic phase removal [41]. Goldstein filtering was applied to all the interferograms to reduce the signal noise and Delaunay 3D method was used for phase unwrapping with a coherence threshold of 0.25. In the **refinement and re-flattening** stage, unwrapped interferometric phases were refined and re-flattened using the polynomial method to estimate and remove the remaining phase constants and phase ramps based on the residual phase method. For this stage, 65 ground control points were selected with rational distribution on the unwrapped phase based on the following spatial location suitability criteria: no high frequency residual topography fringes, no phase jumps corresponding to unwrapping errors or no displacement fringes, also meaning collection was far from a displacement area [42,43,44]. Further on, the **first inversion** step was performed to derive the residual height and the displacement velocity to flatten the complex interferograms by re-calculating the phase unwrapping. The **second inversion** was performed to derive filtered displacements with removed atmospheric phase components. **SBAS geocoding** was performed to produce geo-references velocities and displacements in the satellite line-of-sight (LOS) direction, deriving the final products in both vector and raster formats. It is well known that InSAR does not directly measure vertical and horizontal displacements but rather measures their projection along the LOS direction, for DSC and ASC tracks, according to the data acquisition geometry, which can be decomposed along the vertical and horizontal directions [45,46]. Therefore, the LOS velocities derived from ASC and DSC tracks of CSK, TSX and SNT1 datasets were separately decomposed into the horizontal component along the east-west direction, *d*_hor_, and the vertical component, *d*_ver_, taking into account the local incidence angle of the satellite view by the Equation (1) [47,48,49,50,51,52,53].

(1) $$\left(\begin{array}{c} d_{\mathrm{asc}}\\ d_{\mathrm{dsc}} \end{array}\right)\;=\;\left(\begin{array}{c} \cos\theta_{\mathrm{asc}}-\cos\alpha_{\mathrm{asc}}\sin\theta_{\mathrm{asc}}\\ \cos\theta_{\mathrm{dsc}}-\cos\alpha_{\mathrm{dsc}}\sin\theta_{\mathrm{dsc}} \end{array}\right)\left(\begin{array}{c} d_{ver}\\ d_{hor} \end{array}\right)$$

where θ_asc_ and θ_dsc_ are the local incidence angles and α_asc_ and  α_dsc_ are the satellite heading angles of ASC and DSC modes [53].

Due to the availability of only one satellite pass for the TSX data, this dataset was combined with the LOS measurements derived from the CKS ASC dataset since both satellite missions are characterized by a common wavelength and spatial resolution.

The produced LOS, vertical and horizontal displacements from the CSK, TSX and SNT1 satellite images were interpolated using the geostatistical ordinary Krigging method to generate gridded surfaces without spatial gaps. This geostatistical interpolation method contributed to a simplified interpretation and comparative geospatial overlay data analytics.

The location of the injection and extraction wells was identified from the high-resolution Worldview-3 optical acquisitions. The wells have been overlaid with the results obtained from the SAR interferometric analysis to search for possible relationships with the vertical and horizontal components of deformation.

## 4. Results

**Figure 5:**
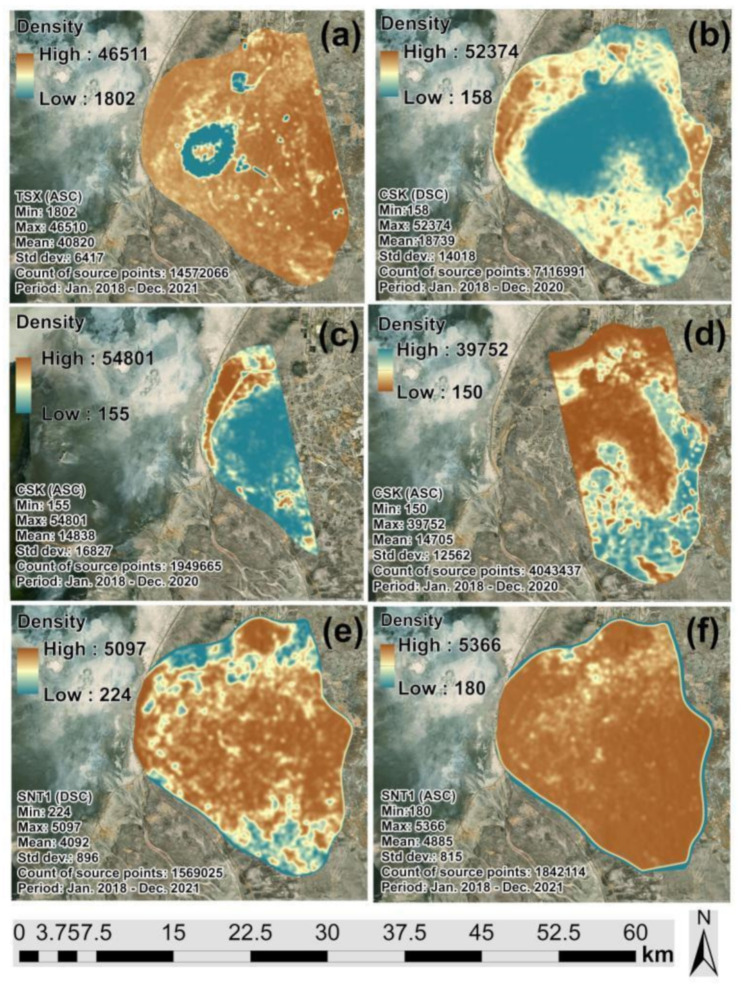
Kernel density grids of SBAS-InSAR measurements using:(**a**) TSX (ASC Track); (**b**) CSK (DSC Track); (**c**) CSK (ASC Track 1); (**d**) CSK (ASC Track 2); (**e**) SNT1 (DSC Track); (**f**) SNT1 (ASC).

Figure 5a–f present the kernel density grids developed based on the SBAS-InSAR measured points from the TSX, CSK and SNT1 images, respectively. It is possible to observe that SBAS-InSAR generated significantly higher point density for the TSX than CSK, considering these missions are characterized by a common spatial resolution and wavelength. As expected, SNT1 point density is much lower, as the spatial resolution of this dataset is much lower. These differences are reflected in the **Min** values of kernel density grids since the highest value of 1802 was observed for the TSX images (Figure 5a–f). The standard deviation of measurements from the TSX and SNT1 images indicated lower variation and more rational distribution over the Tengiz oilfield than CSK (Figure 5a–f).

**Figure 6:**
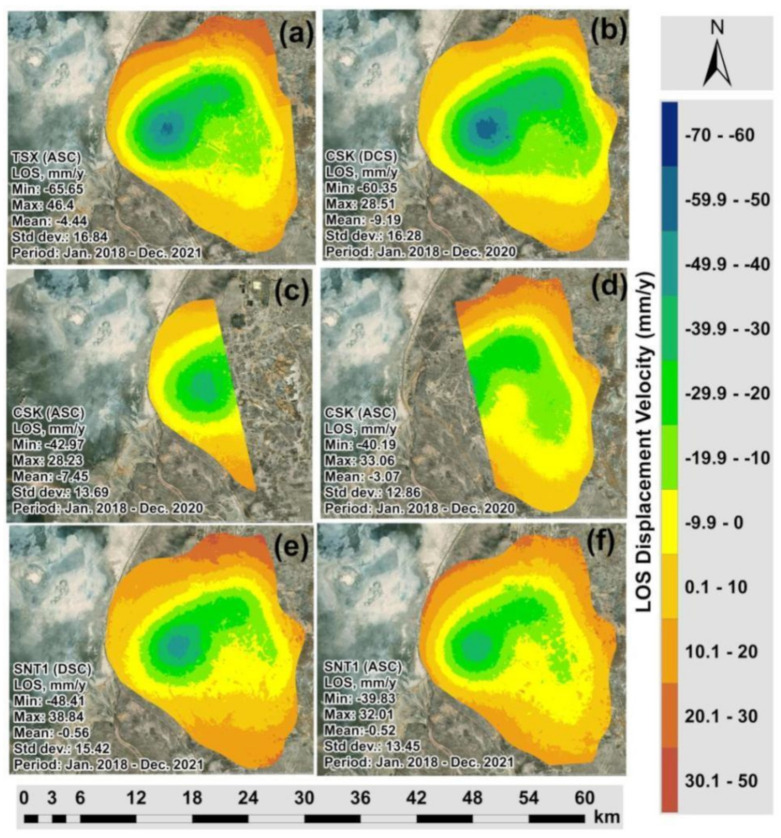
Maps of LOS vertical displacement velocity of the Tengiz oilfield derived from: (**a**) TSX (ASC Track); (**b**) CSK (DSC Track); (**c**) CSK (ASC Track 1); (**d**) CSK (ASC Track 2); (**e**) SNT1 (DSC Track); (**f**) SNT1 (ASC).

**Figure 7:**
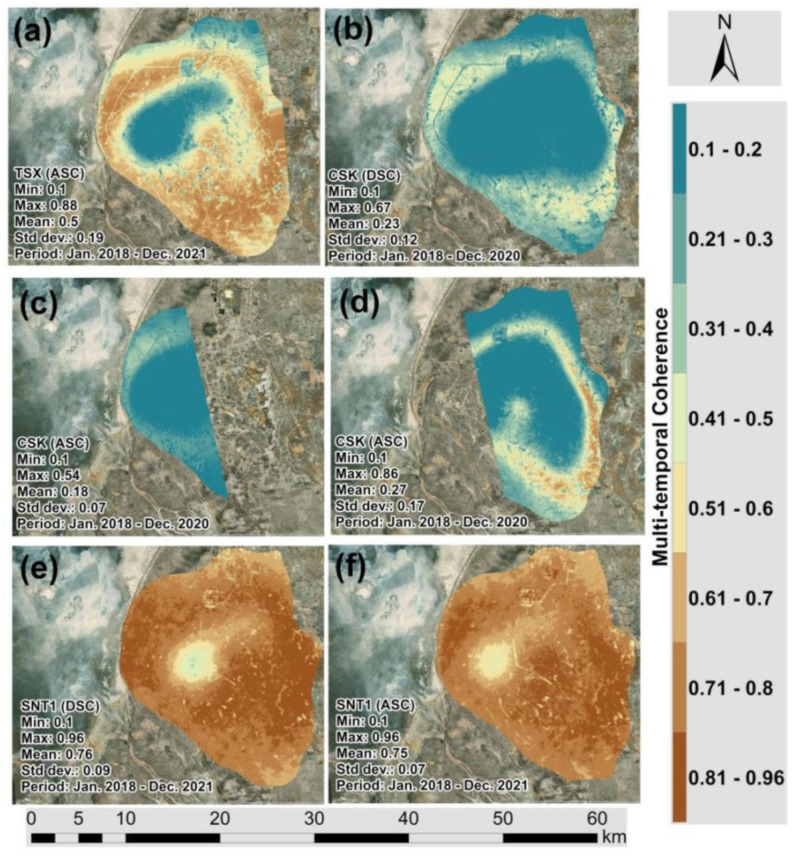
Multi-temporal coherence: (**a**) TSX (ASC Track); (**b**) CSK (DSC Track); (**c**) CSK (ASC Track 1); (**d**) CSK (ASC Track 2); (**e**) SNT1 (DSC Track); (**f**) SNT1 (ASC).

**Figure 8:**
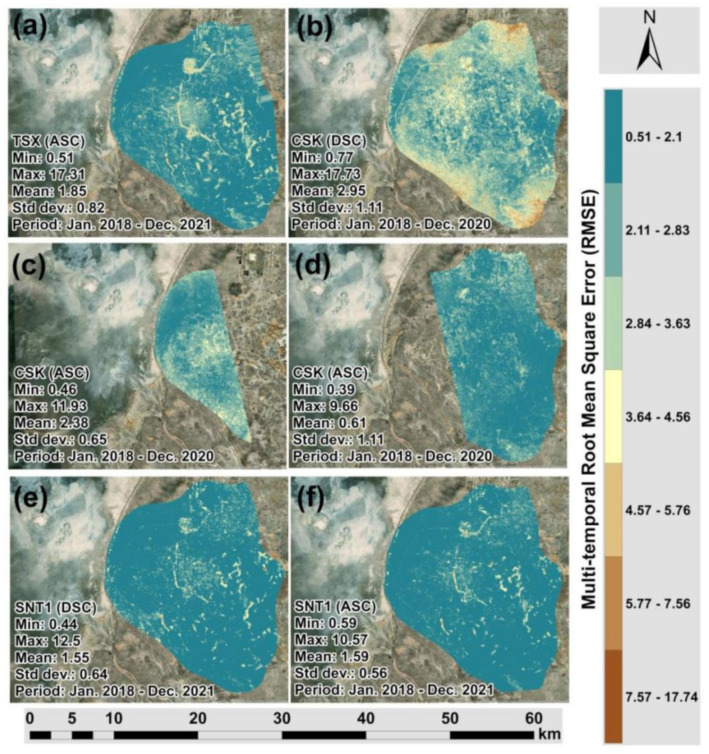
Multi-temporal Root Mean Square Error: (**a**) TSX (ASC Track); (**b**) CSK (DSC Track); (**c**) CSK (ASC Track 1); (**d**) CSK (ASC Track 2); (**e**) SNT1 (DSC Track); (**f**) SNT1 (ASC).

Figure 6a–f present the LOS surface deformations derived from TSX, CSK and SNT1 datasets, respectively. It is possible to clearly observe that the subsidence patterns indicated by green and blue color classes are localized in the center of the Tengiz oilfield. Comparing the kernel density grids (Figure 5a–f) and LOS displacement measurements (Figure 6a–f), the major coverage of low-density measurements from CSK images are located within the subsiding area. It is possible to conclude that CSK images showed higher decorrelation over time and space for the period of 2018–2020. On the other hand, the count of TSX and SNT1 images used in the SBAS-InSAR processing for 2018–2021 was higher than the count of CSK images (Table 1). This fact could also contribute to the higher density of points measured by SBAS-InSAR using TSK and SNT1 images. Furthermore, Figure 7a showed significantly higher multi-temporal coherence for TSX compared to CSK images in Figure 7b–d. SNT1 also showed significantly higher multi-temporal coherence than CSK images Figure 7e,f. This was reflected in the larger spatial coverage of higher multi-temporal coherence for TSX and SNT1 compared to CSK. Multi-temporal coherence with **Max** = 0.88 and **Mean** = 0.5 for TSX and **Max** = 0.96 and **Mean** = 0.76 for SNT1 was observed to be significantly higher than in case of CSK (Figure 7a,e,f). Multi-temporal Root Mean Square Error (RMSE) grids are presented in Figure 8a–f. Larger spatial coverage of higher multi-temporal RMSE was observed for CSK in Figure 8b,c. In fact, multi-temporal RMSE with **Mean** = 2.95 in Figure 8b and **Mean** = 2.38 in Figure 8c of CSK were observed to be higher than for TSX and SNT1. Lower multi-temporal coherence and higher RMSE of CSK allowed us to conclude that a factor of decorrelation ending with lower SBAS-InSAR point density for CSK is not driven by a lower quantity of processed CSK images than TSX and SNT1. In addition, it is necessary to emphasize that 87 CSK DSC Images (Figure 2) were considered to be sufficient for the SBAS-InSAR processing. The studies by Baghdadi et al. [54] and Pettinato et al. [55] performed a comparison between TSX and CSK SAR sensors and reported the stability of backscattering SAR signals and a higher backscattering coefficient for TSX than CSK. The studies by Mora et al. [56] demonstrated a good agreement of produced interferometric results derived from CSK and SNT1 images. Although both satellite missions have different spatial resolution and wavelengths, Mora et al. 2017 detected ground motion patterns with similar precisions.

**Figure 9:**
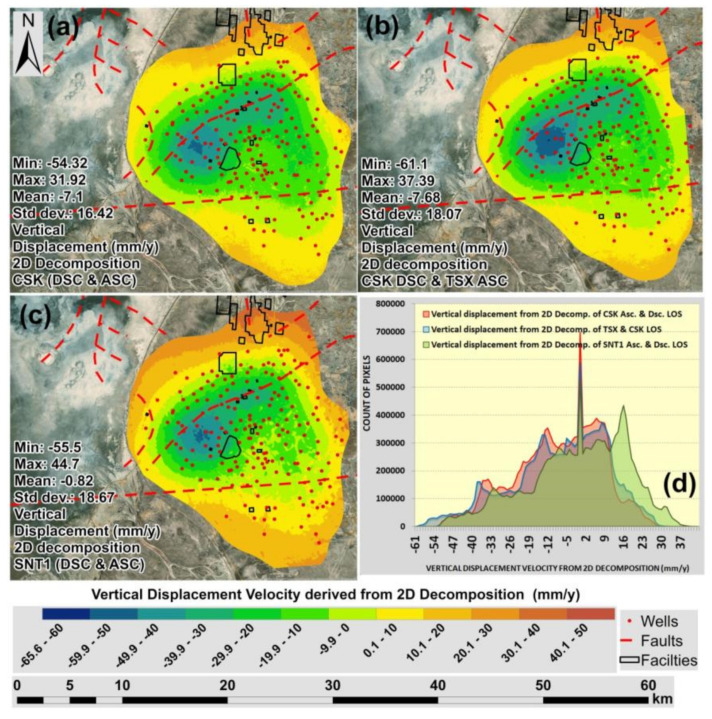
Maps of vertical displacement velocity (2018–2020) derived from 2D decompositions of: (**a**) CSK DSC/ASC Tracks; (**b**) CSK DSC/TSX ASC Tracks; (**c**) SNT1 DSC/SNT1 ASC Tracks; (**d**) Histograms of vertical displacements.

The maps of vertical displacement velocities derived from the 2D decomposition of CSK DSC/CSK ASC tracks, CSK DSC/TSX ASC tracks and SNT1 DSC/SNT1 ASC tracks are presented in Figure 9a–c. As it is possible to observe, they all showed similar ground motion patterns with primary subsidence towards the central area of the Tengiz oilfield. The highest rate of subsidence velocity detected from three satellite missions was observed to be in the range between −61 mm/year and −54 mm/year. As it is possible to observe in the histogram, which is presented in Figure 9d, the maximum subsidence was derived from the 2D decomposition of LOS measurements from CSK DSC/TSX ASC tracks. It is also reflected in the CSK DSC/TSX ASC tracks’ vertical displacement value of **Mean** = −7.68 mm/year which is lower than CSK DSC/CSK ASC and SNT1 DSC/SNT1 ASC. The **standard deviation** was observed to be similar for the three satellite missions which means that there are no overall significant variations in the vertical displacements derived from SBAS-InSAR measurements and 2D decompositions.

**Figure 10:**
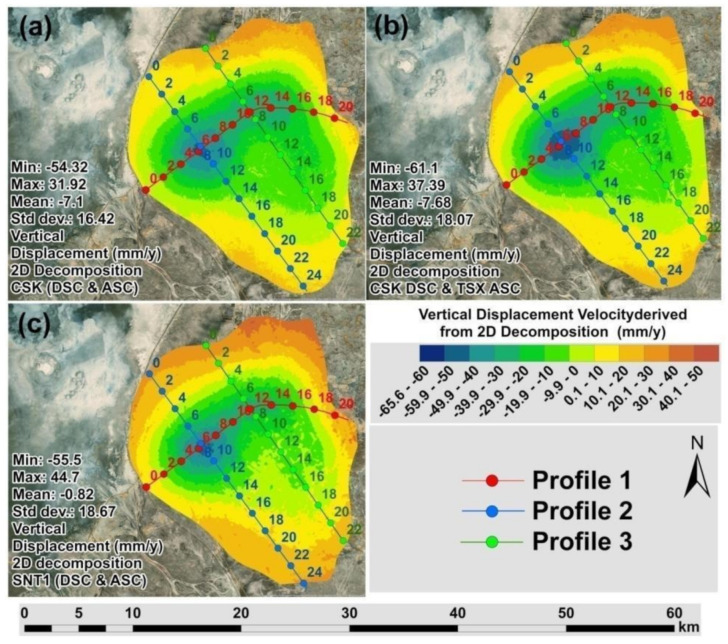
Maps with profiles of vertical displacement velocity (2018–2020) derived from 2D decompositions of: (**a**) CSK DSC/CSK ASC Tracks; (**b**) CSK DSC/TSX ASC Tracks; (**c**) SNT1 DSC/SNT1 ASC Tracks.

**Figure 11:**
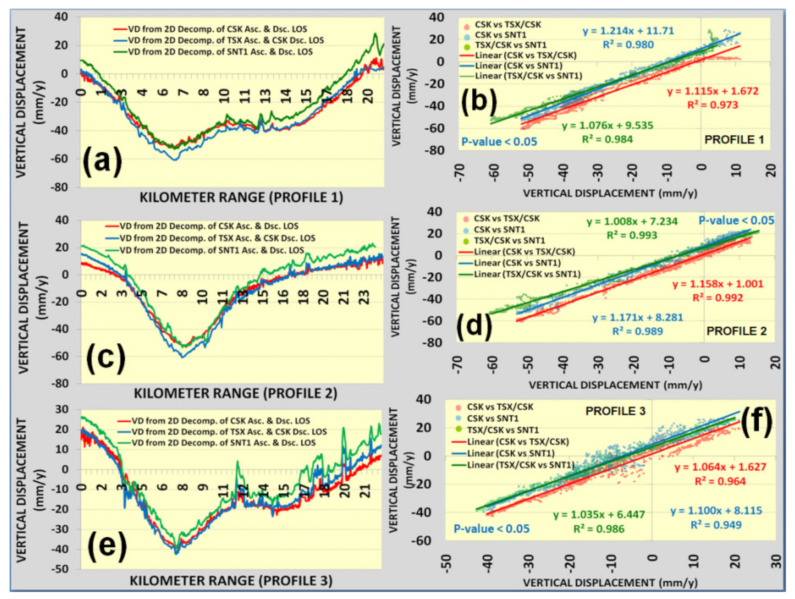
Profiles of vertical displacement velocities: (**a**) profile 1; (**b**) regression analysis for profile 1;(**c**) profile 2; (**d**) regression for profile 2; (**e**) profile 3; (**f**) regression analysis for profile 3.

**Figure 12:**
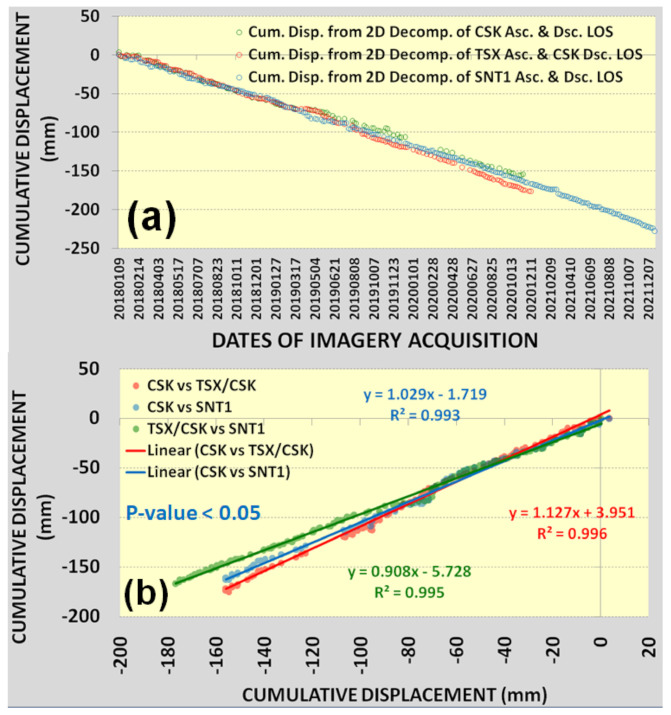
(**a**) Cumulative vertical displacements for the detected maximum subsiding location (intersection of profile 1 & profile 2); (**b**) regression for cumulative vertical displacements.

The profile lines crossing the detected subsidence hotspots were analyzed to assess correlations between measured vertical displacement velocities (Figure 10a–c). Vertical displacement velocities derived from 2D Decomposition of CSK DSC/CSK ASC, CSK DSC/TSX ASC and SNT1 DSC/SNT1 ASC measurements showed a good agreement with an average regression coefficient of 0.98 (Figure 11a–f). Apart from the vertical displacements, 2D decomposition of LOS measurements from different satellite missions played a significant role in the correct alignment of SBAS-InSAR measurements in the vertical and horizontal space and consequently contributed to the relative analyses against each other. Direct analysis of LOS measurements by petroleum and gas operators may be misleading because they are relative to descending and descending tracks rather than actual vertical displacement (Figure 6a–f). It is also necessary to emphasize that the projected horizontal positioning differences existing in the LOS measurements may be misleading to accurately determine subsidizing or uplifting positions on the ground for the assessment and mitigation of ground deformation risks (Figure 6a–f). The highest rate of subsidence in the Tengiz oil field is located at the intersection of Profile 1 and Profile 2 in Figure 10a–c. The cumulative displacements showed a good agreement for the most subsiding location with an average regression coefficient of 0.99 (Figure 12a,b).

**Figure 13:**
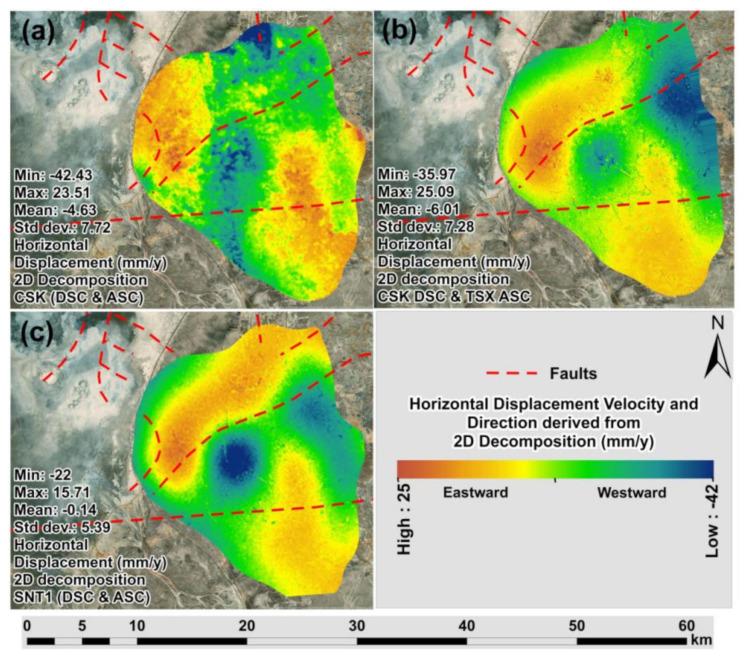
Maps of horizontal displacement velocity (2018–2020) derived from 2D decompositions of: (**a**) CSK DSC/CSK ASC tracks; (**b**) CSK DSC/TSX ASC tracks; (**c**) SNT1 DSC/SNT1 ASC tracks.

**Figure 14:**
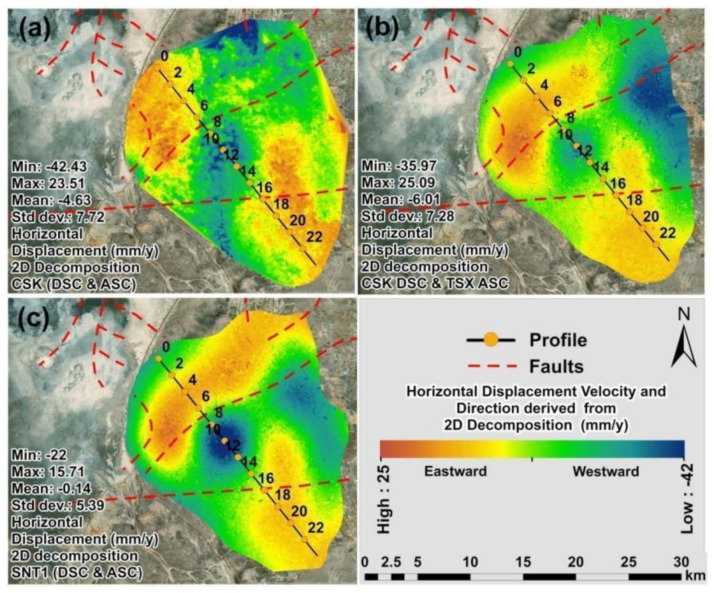
Maps with profiles of horizontal displacement velocity (2018–2020) derived from 2D decompositions of: (**a**) CSK DSC/CSK ASC tracks; (**b**) CSK DSC/TSX ASC tracks; (**c**) SNT1 DSC/SNT1 ASC tracks.

**Figure 15:**
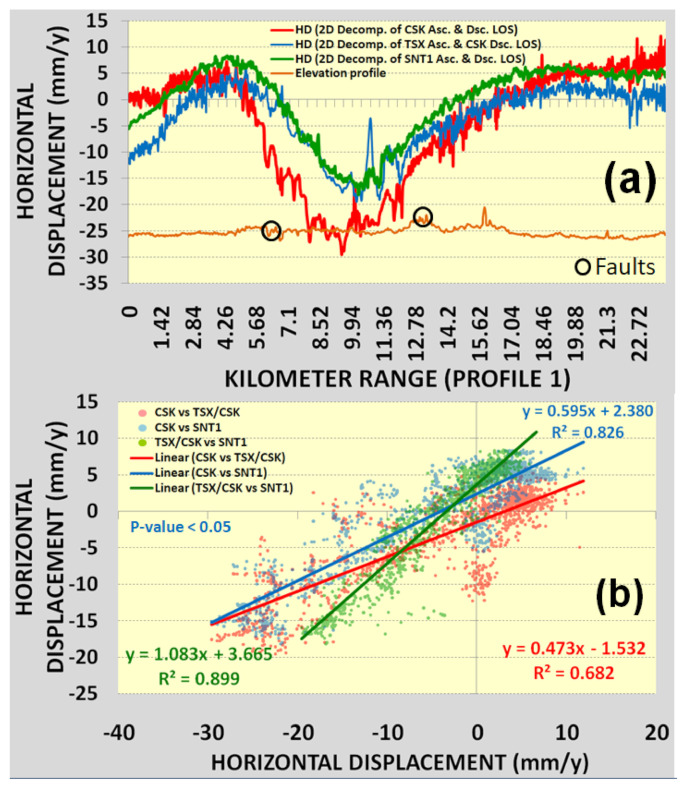
Horizontal displacement velocity: (**a**) profile; (**b**) regression for profile.

The maps of horizontal displacement velocities derived from the 2D decomposition of CSK DSC/CSK ASC tracks, CSK DSC/TSX ASC tracks and SNT1 DSC/SNT1 ASC tracks are presented in Figure 13a–c. Comparing the different datasets combinations, higher variations in ground motion patterns were observed for horizontal than vertical displacement velocities. These variations were clearly represented in the profile line indicated in Figure 14a–c and Figure 15a,b with an average regression coefficient of 0.80 (Figure 15b). The lowest regression coefficient of 0.68 was observed for the horizontal displacement velocities derived from CSK DSC/CSK ASC and CSK DSC/TSX ASC tracks.

**Figure 16:**
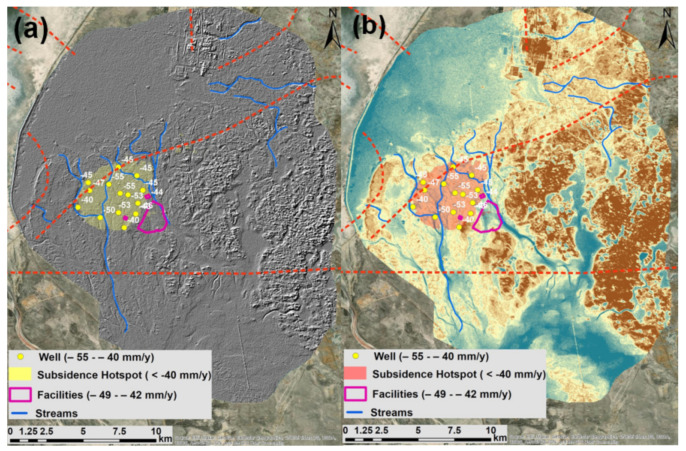
(**a**) Terrain Hillshaded representation with Wells and Facilities; (**b**) DEM with Wells and Facilities falling between −55.6 mm/year and −42 mm/year.

**Figure 17:**
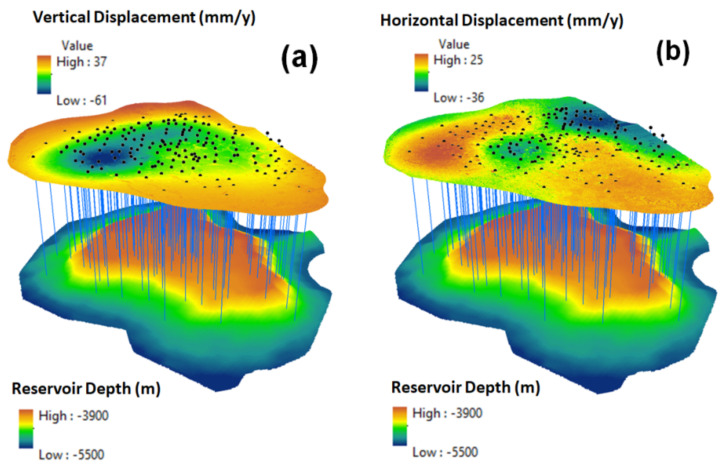
3D models of: (**a**) vertical displacement velocity and reservoir depth; (**b**) horizontal displacement velocity and reservoir depth.

Figure 16a,b presents fifteen wells and three facilities that were observed to be within the subsidence hotspot. They are located within the subsidence range between −55.6 mm/year and −42 mm/year. Based on Figure 16a,b, it is possible to observe two seismic faults from the southern and northern sides, decreasing elevation changes from south to north and forming water streams. The depth of the reservoir’s upper limit and vertical and horizontal displacements are presented in Figure 17a,b, respectively.

## 5. Discussion

In this study, we showed that vertical and horizontal east-west motion can be derived through the 2D decomposition of SBAS-InSAR LOS measurements from CSK, TSX and SNT1 SAR images. The relative comparison of interferometric measurements derived from CSK, TSX and SNT1 images indicated a good agreement with a strong correlation for vertical displacement velocities and a moderate correlation for horizontal displacement velocities. The results showed that all three satellite missions could provide useful information for identifying spatial deformation patterns and subsidence and uplift hotspots based on the precise interferometric measurements with a high level of reliability and accuracy. Two-dimensional decomposition of LOS measurements to vertical and horizontal displacements is the solution to eliminate variations in ground deformation measurements by different radar satellite missions or direct use of relative LOS measurements for risk assessment and mitigation in the oil and gas industry.

The estimated spatial subsidence patterns were found to be consistent with previous research papers which used different SAR satellite missions and interferometric processing techniques [16,19,20,21,22]. In the studies of Del Conte et al. [16], the maximum annual subsidence velocity was reaching −18 mm/year for the observation period of 2004–2007 (ENVISAT and RADARSAT-1 satellite missions) in the Tengiz oilfield using 2D decomposition techniques to derive vertical and horizontal displacements. In the studies of Comola et al. [19], the maximum annual subsidence velocity was reaching −20 mm/year for the observation period of 2004–2007 (ENVISAT and RADARSAT-1 satellite missions) using a 2D decomposition technique. The studies by Grebby et al. [20] and Orynbassarova [21] showed that the maximum annual subsidence velocity was reaching −16 mm/year during 2004–2009 (ENVISAT) and −79.3 mm/year during 2016–2017 (Sentinel-1) using cosine correction techniques. In the studies by Bayramov et al. [22,23], the maximum annual subsidence velocity was reaching −73.29 mm/year and −77.4 mm/year during 2018–2020 (SNT1 and CSK) using 3D-2D decomposition and cosine correction techniques. All of these studies clearly presented the continuous and increasing subsidence trends in the Tengiz oilfield starting from 2004 till 2020. However, it is difficult to make conclusions about actual vertical and horizontal velocities and cumulative displacements in the Tengiz oilfield since different interferometric processing, LOS to vertical and horizontal displacement techniques and satellite missions were applied.

In the present studies, the observed subsidence hotspot was reaching a vertical deformation velocity up to −54.32 mm/year for CSK DSC/CSK ASC, −61.1 mm/year for CSK DSC/TSX ASC and −55.5 mm/year for SNT1 DSC/SNT1 ASC decompositions during 2018–2021. This allows us to conclude that the application of 2D decomposition to derive vertical and horizontal displacements provided lower variability and deviation in the interferometric measurements of subsidence using three satellite missions. The feasibility, effectiveness and reliability of subsidence estimates by X-band and C-band SAR images for the Tengiz oilfield were clearly observed from the cross-validation regression analysis with the resulting R^2^ higher than 0.95 for the vertical displacement derivatives. It is necessary to emphasize that the vertical displacement derived from the 2D decomposition of LOS measurements by CSK DSC and TSX ASC tracks showed a good agreement with individual CSK and SNT1 decompositions. This means that the oil field operators can benefit from the combination of different satellite SAR images with similar spatial resolution in case either descending or ascending tracks are not available within the same satellite mission. However, this study showed a weaker relationship for horizontal east-west velocities derived from three satellite missions (R^2^ = 0.80).

CSK images showed higher multi-temporal decorrelation and RMSE over time and this resulted in lower measured point density, in particular for the subsiding areas of the Tengiz oilfield. SBAS-InSAR measurements produced twice more point clouds from TSX than CSK. Since SNT1 holds three times lower resolution than CSK, SBAS-InSAR measurements from SNT1 produced an identical count of points like CSK but with better rational spread over the Tengiz oilfield. Produced point cloud density from different satellite missions is a primary requirement in the 2D decomposition of their LOS measurements to obtain vertical and horizontal displacements. In fact, the 2D decomposition can effectively calculate the projected deformation components only where both ASC and DSC datasets are providing results.

In our study, fifteen wells and three facilities were observed to be located within the subsidence range between −55.6 mm/year and −42 mm/year. However, we could not make a proper judgment about the criticality of these displacement rates for the safe operations of oilfields and mitigation of geohazards and geotechnical risks.

The studies by Tamburini et al. [3], Brew et al. [57] and Leezenberg et al. [58] recommended that the contributing role of interferometric measurements depended on the reservoir depth. Since the upper limit Tengiz reservoir depth starts from around −3900 m depth, the actual cause of these ground deformations should be investigated in-depth using subsurface geological and geophysical information to better understand the continuous subsidence processes in this field.

Mahajan et al. [59] studied the impacts of ground deformation related to fault reactivations which resulted in well and facility damages in Oman. The present studies also considered two seismic faults located in the southern and northern parts of detected subsidence hotspots and also the terrain factor with gradually decreasing elevation changes from south to north forming water streams which obviously affect the soil moisture and texture, making them more susceptible to subsidence processes. Therefore, it is plausible to state that, apart from the key oil extraction and injection effects, this subsidence hotspot is also caused by natural tectonic and hydrological processes. This assumption is also based on the fact that there is no higher well density than in other areas of Tengiz oilfield. However, it was not possible to assess the direct negative impact of these two seismic faults on potential damages to oil and gas infrastructure in the Tengiz oilfield.

The detected east–west deformation patterns of the Tengiz oilfield exhibited a possible correlation with the seismic faults’ location and shape, suggesting that the measured horizontal deformations could be affected by natural tectonic processes. Therefore, apart from the impact of the oil extraction and injection activities causing ground deformation, it is also critical to consider natural factors accelerating the subsidence processes in the Tengiz oil field. Nagel et al. [60] concluded that reservoir compaction and subsidence were not always negative but could also contribute to increased production and recovery. Therefore, the role of the present study is not limited only to geohazard and geotechnical surveillance but also plays a crucial role in the detailed studies of oil and gas reservoirs’ dynamic behaviors.

The application of CSK, TSX and SNT1 images should be selected depending on the scale of analysis, required density and precision of measurements. However, especially in case of the high-resolution CSK and TSX images, it is highly recommended to limit the analysis redundancy by considering a limited number of connections and temporal baselines for the generation of optimized number of interferograms. For operational monitoring, this will allow for the balancing of the acceptable speed of processing and the required quality and accuracy of interferometric measurements.

SBAS-InSAR surface displacements coupled with the geospatial and geostatistical analyses can significantly improve our understanding of reliability, quality and differences of interferometric measurements derived from various satellite missions and also applied processing techniques. Additionally, geospatial analytics significantly contribute to the understanding of active tectonic and seismicity mechanisms driving subsidence processes.

The present study also demonstrated the importance of retrieving vertical and horizontal components of deformations, overcoming the direct use of LOS measurements, to additionally support the interpretation of InSAR results by petroleum and gas operators. In the present research, the differences between the LOS measurements and vertical displacements were observed to be in the range of 10–20 mm. The horizontal positioning differences of LOS measurements derived from CSK, TSX and SNT1 satellite missionswere quite indicative of the relative location of subsidence hotspots. Considering the fact that oil and gas industries use accurate positioning and reliable subsidence velocities and cumulative rates to plan investments into the in-situ remediation measures, it is crucial to supply the most precise measurements to justify the needed costs for risk mitigation. Precise measurements are also important to make reliable decisions regarding the criticality of subsidence rates against defined organizational engineering standards [22]. Fuhrmann et al. [61] also detected maximum vertical motion displaced horizontally by several hundred meters when the LOS measurements were just projected for a study area in Australia. Fuhrmann et al. [61] clearly stated that vertical measurements derived from LOS projection could be wrong and misleading in terms of magnitude, location and direction and recommended the fusing of LOS measurements from several different viewing geometries for the achievement of reliable vertical and horizontal displacements.

However, the present research results also exhibit the shortcomings of GPS ground measurements; therefore, an alternative approach was used to perform cross-validation among the results from the three satellite missions. It is planned to continue the present study by integrating in-situ historical geodetic measurements and more subsurface data from oil and gas operators of the Tengiz oilfield to more accurately validate current InSAR measurements and also better understand subsidence controlling subsurface factors. It is also planned to apply coherent change detection analyses for backscattering properties of radar images to better understand historical, natural and man-made changes over the Tengiz oilfield [62].

As an enhancement for the present studies, it is planned to apply machine and deep learning techniques for the optimization, simplification and quality enhancement of surface displacement measurements and analyses in the Tengiz oilfield. For instance, as a limitation of the present studies, it is necessary to enhance the present studies with machine learning techniques in particular to resolve spatial discontinuity issues. The studies by Naghibi et al. 2022 applied integrated InSAR-machine learning to address challenges related to spatial discontinuity due to the vegetation decorrelation [63]. The studies by Brengman et al. 2021 used the Convolutional Neural Networks (CNNs) to detect, locate, and classify co-seismic surface deformation within synthetic interferograms [64].

Apart from the present studies for the selected oil field with known vulnerabilities to deformation processes, similar subsidence processes might be subject to other oil and gas fields in Kazakhstan. Therefore, the application of existing machine and deep learning practices may play a significant role in the analyses of big microwave data to detect the majority of subsiding petroleum and gas fields in Kazakhstan. Anantrasirichai et al. 2019 explored the feasibility and limits of CNNs for detecting slow, sustained deformations in wrapped interferograms [65]. The studies by Anantrasirichai et al. 2020 explored the applicability of deep learning approaches by adapting pre-trained CNNs for the detection of deformations on a national scale [66]. Mukherjee et al. 2018 explored and proposed a CNN-based coherence classifier with less misclassification in incoherent areas than existing methods for InSAR images [67]. Liu et al. 2022 automated the recognition of landslides using InSAR images and the improved mask R-CNN Model [68].

In addition, it is also planned to analyze multi-spectral and hyperspectral optical images to measure temporal changes in other surface properties like temperature, moisture, etc., for the Tengiz oilfield.

## 6. Conclusions

Vertical displacement velocities derived from the 2D decomposition of CSK DSC/CSK ASC, CSK DSC/TSX ASC and SNT1 DSC/SNT1 ASC measurements showed a good agreement in similar ground motion patterns with an average regression coefficient of 0.98. The average maximum vertical subsidence from three satellite missions was observed to be −57 mm/year. Higher variations and deviations were observed for horizontal displacement velocities in terms of similar ground motion patterns and an average regression coefficient of 0.80.

CSK showed lower multi-temporal coherence and higher RMSE than TSX and SNT1. This means that the de-correlation factor of CSK images over 2018–2020 was significantly higher than TSX and SNT1. This was reflected in twice the lower point density produced from CSK than from TSX and SNT1 in particular for the subsiding areas of the Tengiz oilfield.

The 2D decomposition of CSK DSC/TSX ASC LOS measurements showed a good agreement with CSK DSC/CSK ASC and SNT1 DSC/SNT1 ASC with similar ground motion patterns and an average regression coefficient of 0.98 for vertical and 0.80 for horizontal displacement velocities. Even though TSX showed higher multi-temporal coherence and lower RMSE, its coverage of the Tengiz oilfield from the DSC track was limited. Hence, it is feasible for Tengiz oilfield to combine different SAR satellite missions of similar resolution when either DSC or ASC tracks are missing.

Fifteen wells and three facilities were observed to be located within the subsidence range between −55.6 mm/year and −42 mm/year. However, there is no openly accessible data or clearly defined standards to evaluate whether these deformation rates should be considered critical for the safe operations of oilfields and for the mitigation of geohazards and geotechnical risks.

It is possible to clearly observe that the distribution density of wells is not spatially related to the detected subsidence hotspots. It is also possible to conclude that the amount of extracted oil and applied injection technologies are some of the key factors controlling subsidence processing in the Tengiz oilfield. As the secondary key factor, it is necessary to emphasize the impact of seismic faults which are located in the northern and southern parts of the detected vertical and horizontal displacement velocity hotspots. The detected horizontal movements of the Tengiz oilfield could be additionally affected by natural tectonic processes. As the third key factor, it was possible to observe the local geo-morphology, showing a trend from south to north with natural water streams flowing towards the subsidence hotspots. This obviously changed both the soil structure and moisture content, making it prone to subsidence processes.

Even though LOS measurements and cosine correction approaches are often used by petroleum and gas operators, these studies once again demonstrated the importance of retrieving at least 2D information to obtain horizontal and also vertical positional accuracy to better describe subsidence evolution and dynamics.

Cumulative vertical displacements derived from 2D Decomposition of CSK DSC/CSK ASC, CSK DSC/TSX ASC and SNT1 DSC/SNT1 ASC measurements showed a good agreement for the most subsiding location with an average regression coefficient of 0.99.

Achievements of the present research includea comparative analysis of vertical and horizontal surface displacements derived from three satellite missions and a better understanding of their technical capacities for the reliable monitoring of oil and gas reservoirs.

## Figures and Tables

**Table 1:** Characteristics of SAR images used for the present research [31,32].

Sensor Imaging Mode	Track	Resolutions: Range Azimuth [m × m] &Swath [km]	Revisit Time (Days)	Count of Images	Temporal Span	Polarization Used	Interferometric Mode	Wavelength
TerraSAR-X (TSX)	ACS	3 × 3; 30	11	119	1 January 2018 and 31 December 2021	HH	StripMap	X-band (3 cm, 9.65 GHz)
Cosmo-SkyMED (CSK) Stripmap mode	DSC	3 × 3; 40	4	87	1 January 2018 and 31 December 2020	HH	StripMap HIMAGE	X-band (3.1 cm, 9.6 GHz)
	ASC			28				
				60				
Sentinel-1 (SNT1)	DSC	5 × 20; 250	6	117	1 January 2018 and 31 December 2021	VV	Interferometric Wide Swath (IW) mode	C-band (5.6 cm wavelength and 5.4 GHz)
	ASC			121				
				114				

## Data Availability

Not applicable.

## References

[1] Rabus B., Werner C., Wegmueller U., McCardle A. Interferometric point target analysis of RADARSAT-1 data for deformation monitoring at the Belridge/Lost Hills oil fields. Proceedings of the IEEE Geoscience and Remote Sensing Symposium 2004 (IGARSS’04), Institute of Electrical and Electronics Engineers.

[2] Zhou W., Chen G., Li S., Ke J. InSAR Application in Detection of Oilfield Subsidence on Alaska North Slope. Proceedings of the 41st US Symposium on Rock Mechanics (USRMS).

[3] Tamburini A., Bianchi M., Giannico C., Novali F. (2010). Retrieving Surface Deformation by PS Insar™ Technology: A Powerful Tool in Reservoir Monitoring. Int. J. Greenh. Gas Control.

[4] Ferretti A., Tamburini A., Novali F., Fumagalli A., Falorni G., Rucci A. (2011). Impact of high resolution radar imagery on reservoir monitoring. Energy Procedia.

[5] Gee D., Sowter A., Novellino A., Marsh S., Gluyas J. (2016). Monitoring land motion due to natural gas extraction: Validation of the Intermittent SBAS (ISBAS) DInSAR algorithm over gas fields of North Holland, The Netherlands. Mar. Pet. Geol..

[6] Taylor K., Ghuman P., McCardle A. (2016). Operational mine monitoring with InSAR. Proceedings of the First Asia Pacific Slope Stability in Mining Conference.

[7] Yang C., Zhang D., Zhao C., Han B., Sun R., Du J., Chen L. (2019). Ground Deformation Revealed by Sentinel-1 MSBAS-InSAR Time-Series over Karamay Oilfield, China. Remote Sens..

[8] Benetatos C., Codegone G., Ferraro C., Mantegazzi A., Rocca V., Tango G., Trillo F. (2020). Multidisciplinary Analysis of Ground Movements: An Underground Gas Storage Case Study. Remote Sens..

[9] Bayramov E., Buchroithner M., Kada M., Bayramov R. (2022). Quantitative assessment of ground deformation risks, controlling factors and movement trends for onshore petroleum and gas industry using satellite radar remote sensing and spatial statistics. Georisk Assess. Manag. Risk Eng. Syst. Geohazards.

[10] Staniewicz S., Chen J., Lee H., Olson J., Savvaidis A., Reedy R., Breton C., Rathje E., Hennings P. (2020). InSAR reveals complex surface deformation patterns over an 80,000 km^2^ oil-producing region in the Permian Basin. Geophys. Res. Lett..

[11] Togaibekov A.Z. (2020). Monitoring of Oil-Production-Induced Subsidence and Uplift. Master’s Thesis.

[12] Holloway S., Pearce J.M., Hards V.L., Ohsumi T., Gale J. (2007). Natural emissions of CO_2_ from the geosphere and their bearing on the geological storage of carbon dioxide. Energy.

[13] Xu Y. (2014). Analysis of common geological hazards in oil and gas fields. Heilongjiang Sci. Technol. Inf..

[14] Shi J., Yang H., Peng J., Wu J., Xu B., Liu Y., Zhao B. (2019). InSAR Monitoring and analysis of ground deformation due to fluid or gas injection in Fengcheng oil field, Xinjiang, China. J. Indian Soc. Remote Sens..

[15] Fokker P.A., Visser K., Peters E., Kunakbayeva G., Muntendam-Bos A.G. (2012). Inversion of surface subsidence data to quantify reservoir compartmentalization: A field study. J. Pet. Sci. Eng..

[16] Del Conte S., Tamburini A., Cespa S., Rucci A., Ferretti A. Advanced InSAR Technology for Reservoir Monitoring and Geomechanical Model Calibration. Proceedings of the SPE Kuwait Oil and Gas Show and Conference.

[17] Rocca F., Rucci A., Ferretti A., Bohane A. (2013). Advanced InSAR interferometry for reservoir monitoring. First Break.

[18] Even M., Westerhaus M., Simon V. (2020). Complex Surface Displacements above the Storage Cavern Field at Epe, NW-Germany, Observed by Multi-Temporal SAR-Interferometry. Remote Sens..

[19] Comola F., Janna C., Lovison A., Minini M., Tamburini A., Teatini P. (2016). Efficient global optimization of reservoir geomechanical parameters based on synthetic aperture radar-derived ground displacements. Geophysics.

[20] Grebby S., Orynbassarova E., Sowter A., Gee D., Athab A. (2019). Delineating ground deformation over the Tengiz oil field, Kazakhstan, using the Intermittent SBAS (ISBAS) DInSAR algorithm. Int. J. Appl. Earth Obs. Geoinf..

[21] Orynbassarova E. (2019). Improvement of the Method of Integrated Preparation and Use of Space Images in Tasks of Assessment of Sedimentation of Industrial Surface in the Conditions of Operation of Tengiz Oil and Gas Field. Ph.D. Thesis.

[22] Bayramov E., Buchroithner M., Kada M., Zhuniskenov Y. (2021). Quantitative Assessment of Vertical and Horizontal Deformations Derived by 3D and 2D Decompositions of InSAR Line-of-Sight Measurements to Supplement Industry Surveillance Programs in the Tengiz Oilfield (Kazakhstan). Remote Sens..

[23] Bayramov E., Buchroithner M., Kada M., Duisenbiyev A., Zhuniskenov Y. (2022). Multi-Temporal SAR Interferometry for Vertical Displacement Monitoring from Space of Tengiz Oil Reservoir Using SENTINEL-1 and COSMO-SKYMED Satellite Missions. Front. Environ. Sci..

[24] Zhantaev Z., Fremd A., Ivanchukova A., Kaldybayev A., Nurakynov S., Kantemirov Y., Nikiforov S. (2012). Satellite radar monitoring of land surface subsidence over Tengiz oil and gas field. Geomat. Mag..

[25] Zhantayev Z., Fremd A., Ivanchukova A. Using of SAR data and PSINSAR technique for monitoring of ground deformation in Western Kazakhstan. Proceedings of the 13th International Multidisciplinary Scientific GeoConference SGEM 2013.

[26] Peake W.T., Camerlo R.H., Tankersley T.H., Zhumagulova A. Tengiz Reservoir Uncertainty Characterization and Modeling. Proceedings of the SPE Caspian Carbonates Technology Conference.

[27] Anissimov L., Postnova E., Merkulov O. (2000). Tengiz oilfield: Geological model based on hydrodynamic data. Pet. Geosci..

[28] Imamoglu M., Kahraman F., Çakir Z., Sanli F.B. (2019). Ground Deformation Analysis of Bolvadin (W. Turkey) by Means of Multi-Temporal InSAR Techniques and Sentinel-1 Data. Remote Sens..

[29] Vaka D.S., Sharma S., Rao Y.S. Comparison of HH and VV Polarizations for Deformation Estimation using Persistent Scatterer Interferometry. Proceedings of the 38th Asian Conference on Remote Sensing—Space Applications: Touching Human Lives, ACRS 2017.

[30] Ittycheria N., Vaka D.S., Rao Y.S. Time series analysis of surface deformation of Bengaluru city using Sentinel-1 images. Remote Sensing and Spatial Information Sciences. Proceedings of the 2018 ISPRS TC V Mid-Term Symposium “Geospatial Technology—Pixel to People”.

[31] Tapete D., Cigna F. (2019). COSMO-SkyMed SAR for Detection and Monitoring of Archaeological and Cultural Heritage Sites. Remote Sens..

[32] Yang Y.-J., Hwang C., Hung W.-C., Fuhrmann T., Chen Y.-A., Wei S.-H. (2019). Surface Deformation from Sentinel-1A InSAR: Relation to Seasonal Groundwater Extraction and Rainfall in Central Taiwan. Remote Sens..

[33] Berardino P., Fornaro G., Lanari R., Sansosti E. (2002). A new algorithm for surface deformation monitoring based on small baseline differential SAR interferograms. IEEE Trans. Geosci. Remote Sens..

[34] (2021). Sarmap. SBAS Tutorial. https://www.sarmap.ch/index.php/software/sarscape.

[35] Loesch E., Sagan V. (2018). SBAS Analysis of Induced Ground Surface Deformation from Wastewater Injection in East Central Oklahoma, USA. Remote Sens..

[36] Lanari R., Mora O., Manunta M., Mallorquí J.J., Berardino P., Sansosti E. (2004). A small-baseline approach for investigating deformations on full-resolution differential SAR interferograms. IEEE Trans. Geosci. Remote Sens..

[37] Hooper A., Zebker H.A. (2007). Phase unwrapping in three dimensions with application to InSAR time series. JOSA A.

[38] Tizzani P., Berardino P., Casu F., Euillades P., Manzo M., Ricciardi G.P., Zeni G., Lanari R. (2007). Surface deformation of Long Valley caldera and Mono Basin, California, investigated with the SBAS-InSAR approach. Remote Sens. Environ..

[39] Qiu Z., Jiang T., Zhou L., Wang C., Luzi G. (2019). Study of subsidence monitoring in Nanjing City with small-baseline InSAR approach. Geomat. Nat. Hazards Risk.

[40] Pawluszek-Filipiak K., Borkowski A. (2020). Integration of DInSAR and SBAS Techniques to Determine Mining-Related Deformations Using Sentinel-1 Data: The Case Study of Rydułtowy Mine in Poland. Remote Sens..

[41] Farr T., Rosen P., Caro E., Crippen R., Duren R., Hensley S., Alsdorf D. (2007). The shuttle radar topography mission. Rev. Geophys..

[42] Gaber A., Darwish N., Koch M. (2017). Minimizing the Residual Topography Effect on Interferograms to Improve DInSAR Results: Estimating Land Subsidence in Port-Said City, Egypt. Remote Sens..

[43] Aimaiti Y., Yamazaki F., Liu W., Kasimu A. (2017). Monitoring of Land-Surface Deformation in the Karamay Oilfield, Xinjiang, China, Using SAR Interferometry. Appl. Sci..

[44] Darvishi M., Schlögel R., Kofler C., Cuozzo G., Rutzinger M., Zieher T., Toschi I., Remondino F., Mejia-Aguilar A., Thiebes B. (2018). Sentinel-1 and Ground-Based Sensors for Continuous Monitoring of the Corvara Landslide (South Tyrol, Italy). Remote Sens..

[45] Khorrami M., Abrishami S., Maghsoudi Y., Alizadeh B., Perissin D. (2020). Extreme Subsidence in a Populated City (Mashhad) Detected by PSInSAR Considering Groundwater Withdrawal and Geotechnical Properties. Sci. Rep..

[46] Makabayi B., Musinguzi M., Otukei J. (2021). Estimation of Ground Vertical Displacement in Landslide Prone Areas Using PS-InSAR. A Case Study of Bududa, Uganda. Int. J. Geosci..

[47] Fialko Y. (2006). Interseismic strain accumulation and the earthquake potential on the southern San Andreas Fault system. Nature.

[48] Motagh M., Shamshiri R., HaghshenasHaghighi M., Wetzel H.U., Akbari B., Nahavandchi H., Roessner S., Arabi S. (2017). Quantifying groundwater exploitation induced subsidence in the Rafsanjan plain, southeastern Iran, using InSAR time-series and in situ measurements. Eng. Geol..

[49] Fernandez J., Prieto J.F., Escayo J., Camacho A.G., Luzón F., Tiampo K.F., Mimmo P., Tamara A., Enrique P., Guadalupe P. (2018). Modeling the two- and three-dimensional displacement field in Lorca, Spain, subsidence and the global implications. Sci. Rep..

[50] Aslan G., Cakir Z., Lasserre C., Renard F. (2019). Investigating Subsidence in the Bursa Plain, Turkey, Using Ascending and Descending Sentinel-1 Satellite Data. Remote Sens..

[51] Alatza S., Papoutsis I., Paradissis D., Kontoes C., Papadopoulos G.A. (2020). Multi-Temporal InSAR Analysis for Monitoring Ground Deformation in Amorgos Island, Greece. Sensors.

[52] Minh D.H.T., NGO Y., Lê T.T., Le T.C., Bui H.S., Vuong Q.V., Le Toan T. (2020). Quantifying Horizontal and Vertical Movements in Ho Chi Minh City by Sentinel-1 Radar Interferometry. Preprints.

[53] Aslan G. (2019). Monitoring of surface deformation in northwest Turkey from high-resolution insar: Focus on tectonic a seismic slip and subsidence. Tectonics.

[54] Baghdadi N., El Hajj M., Dubois-Fernandez P., Zribi M., Belaud G., Cheviron B. (2014). Signal Level Comparison Between TerraSAR-X and COSMO-SkyMed SAR Sensors. IEEE Geosci. Remote Sens. Lett..

[55] Pettinato S., Santi E., Paloscia S., Pampaloni P., Fontanelli G. (2013). The Intercomparison of X-Band SAR Images from COSMO-SkyMed and TerraSAR-X Satellites: Case Studies. Remote Sens..

[56] Mora Ó., Pérez-Aragüés F., Marchán J.F., Pipia L. (2017). Sentinel IWS vs. Cosmo-SkyMed Stripmap: A Sensitivity Analysis. Sentinel IWS vs. Cosmo-SkyMed Stripmap: A Sensitivity Analysis. Fringe 2017 Workshop. https://www.icgc.cat/content/download/79789/695658/version/1/file/2017_POSTER_FRINGE_PSI_S1_vs_CSK.pdf.

[57] Brew G.E., Horiuchi M., Leezenberg P.B., Tabak A. Monitoring and Analysis of Surface Deformation with InSAR and Subsurface Data, Sab Joaquin Valley, California. Proceedings of the AAPG Pacific Section and Rocky Mountain Section Joint Meeting.

[58] Leezenberg P.B., Allan M.E. InSAR: Pro-Active Technology for Monitoring Environmental Safety and for Reservoir Management. Proceedings of the SPE Western Regional Meeting.

[59] Mahajan S., Hassan H., Duggan T., Rakesh D. Compaction and Subsidence Assessment to Optimize Field Development Planning for an Oil Field in Sultanate of Oman. Proceedings of the Abu Dhabi International Petroleum Exhibition & Conference.

[60] Nagel N.B. (2001). Compaction and Subsidence Issues within the Petroleum Industry: From wilmington to Ekofisk and beyond. Phys. Chem. Earth A Solid Earth Geod..

[61] Fuhrmann T., Garthwaite M.C. (2019). Resolving Three-Dimensional Surface Motion with InSAR: Constraints from Multi-Geometry Data Fusion. Remote Sens..

[62] Mastro P., Masiello G., Serio C., Pepe A. (2022). Change Detection Techniques with Synthetic Aperture Radar Images: Experiments with Random Forests and Sentinel-1 Observations. Remote Sens..

[63] Naghibi S.A., Khodaei B., Hashemi H. (2022). An integrated InSAR-machine learning approach for ground deformation rate modeling in arid areas. J. Hydrol..

[64] Brengman C.M.J., Barnhart W.D. (2021). Identification of surface deformation in InSAR using machine learning. Geochem. Geophys. Geosyst..

[65] Anantrasirichai N., Biggs J., Albino F., Bull D. (2019). The application of convolutional neural networks to detect slow, sustaineddeformation in InSAR time series. Geophys. Res. Lett..

[66] Anantrasirichai N., Biggs J., Kelevitz K., Sadeghi Z., Wright T., Achim A.M., Bull D. (2020). Detecting Ground Deformation in the Built Environment Using Sparse Satellite InSAR Data With a Convolutional Neural Network. IEEE Trans. Geosci. Remote Sens..

[67] Mukherjee S., Zimmer A., Sun X., Ghuman P., Cheng I. CNN-based InSAR Coherence Classification. Proceedings of the 2018 IEEE SENSORS.

[68] Liu Y., Yao X., Gu Z., Zhou Z., Liu X., Chen X., Wei S. (2022). Study of the Automatic Recognition of Landslides by Using InSAR Images and the Improved Mask R-CNN Model in the Eastern Tibet Plateau. Remote Sens..

